# Supplementary figures and images for: Non-autonomous insulin signaling delays mitotic progression in C. elegans germline stem and progenitor cells
Source: PLoS Genet. 2024 Dec 23;20(12):e1011351. doi: 10.1371/journal.pgen.1011351 (PMC11706408; doi:10.1371/journal.pgen.1011351)

# S1 Figure

A

Number of nuclei in PZ

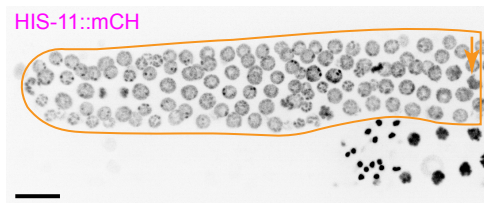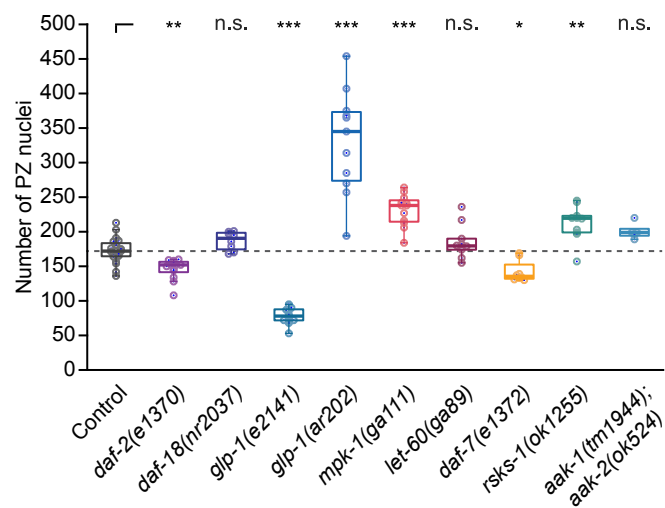

B

Number of cells with 2 centrosomes

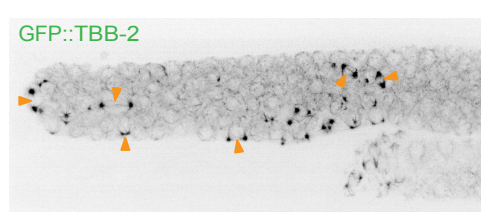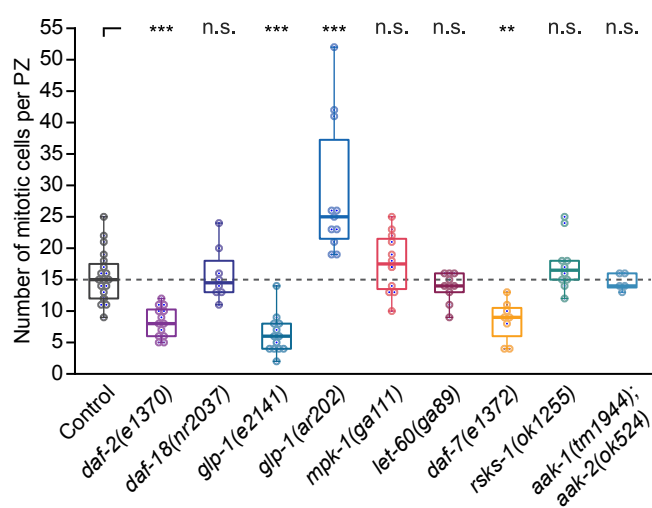

Supplement: S1 Fig — (A) A maximum intensity projection through the top portion of the distal region of one gonad arm from an L4 larva showing germ cell nuclei marked by HIS-11::mCH. The PZ is outlined, with an arrow indicating a crescent-shaped meiotic nucleus marking the proximal end of the PZ. Scale bar = 10 μm. The number of nuclei per PZ used to calculate the mitotic index (Fig 1D) is shown below. (B) A maximum intensity projection through the top portion of the distal region of the same gonad arm shown in (A) showing the GFP::TBB-2 signal. Arrow heads show examples of mitotic spindles in different stages of mitosis (prophase through telophase). The number of mitotic cells per PZ used to calculate the mitotic index (Fig 1D) is shown below. The dashed grey line indicates the median value for control. For all plots, dots represent one PZ and one PZ was assessed per animal. Boxplots show the median, interquartile range and most extreme values not considered statistical outliers. n.s. = p > 0.05, * = p < 0.05, ** = p < 0.01, *** = p < 0.001. Summary statistics and statistical tests used for all figure panels are given in S2 Data. (PDF) [file pgen.1011351.s001.pdf]

**S2 Figure**

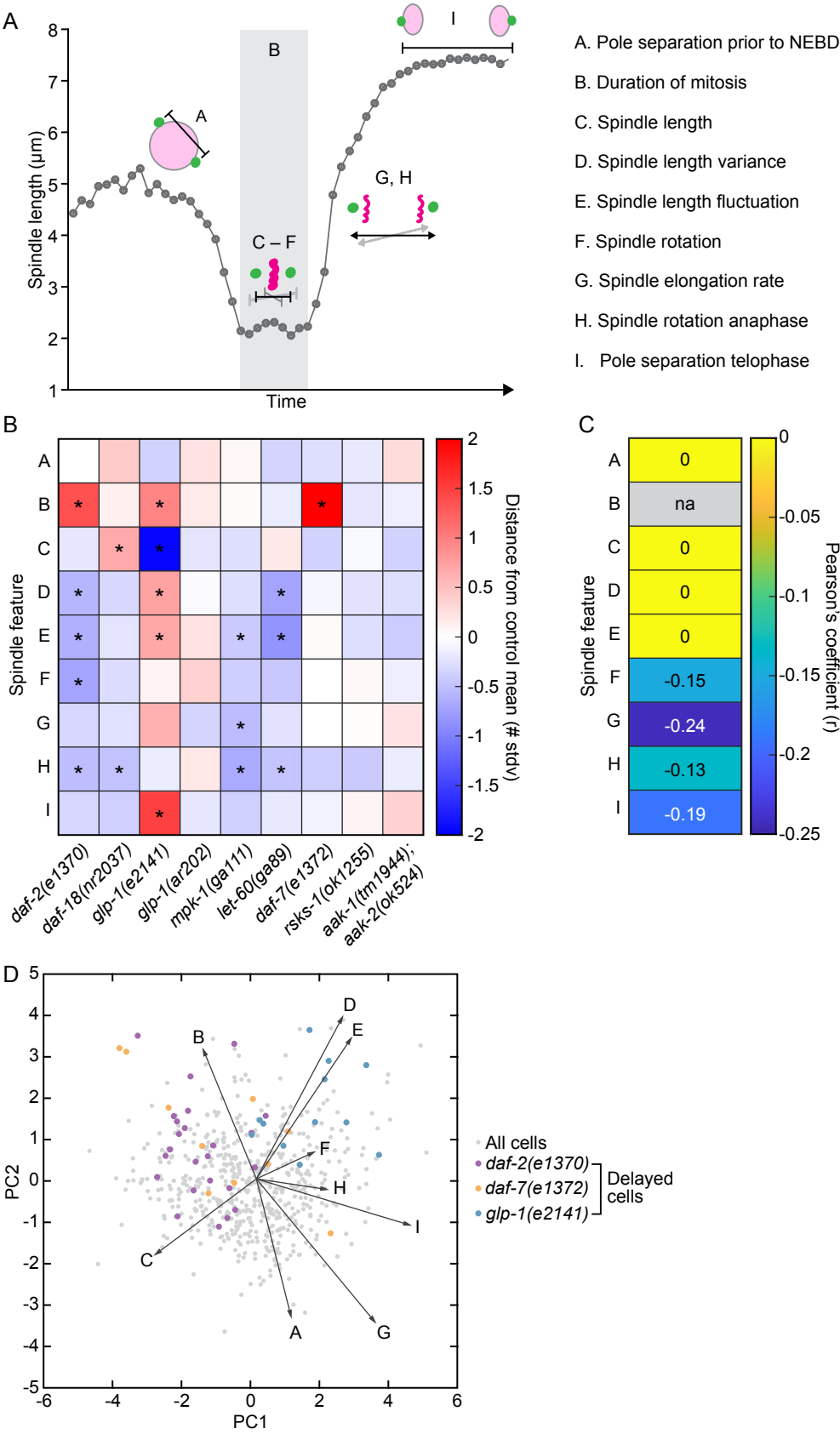

Supplement: S2 Fig — (A) Schematic showing the spindle features extracted for GSPCs from animals bearing mutant alleles in the signaling pathways shown in Fig 1C. Features are shown relative to the spindle length versus time plot from Fig 1B, indicating which stage of mitosis they relate to. Features are listed on the right with letter designations used in (B-D). (B) Heatmap showing the mitotic features that differ significantly from control in each mutant background. Data were compared using a Kruskal-Wallis with Tukey-Kramer post hoc test with the null hypothesis that all samples come from the same distribution. Red indicates values above the control mean and blue indicates values below the control mean. Color saturation indicates the distance above/below the control mean after data standardization. (C) Heatmap showing the strength of the linear relationship between each spindle feature and the duration of mitosis across all cells of all genotypes. Pearson’s coefficient (r) is shown for significant (p < 0.05 after Bonferroni correction) correlations. (D) Principal component analysis of spindle features showing all cells plotted along the first two principal components, with delayed cells (duration of mitosis > 90th percentile of control) shown for daf-2(e1370), daf-7(e1372) and glp-1(e2141). Vectors represent the contribution of each spindle feature to the principal components shown. All data used in this analysis can be found in S1 Data. (PDF) [file pgen.1011351.s002.pdf]

**S3 Figure**

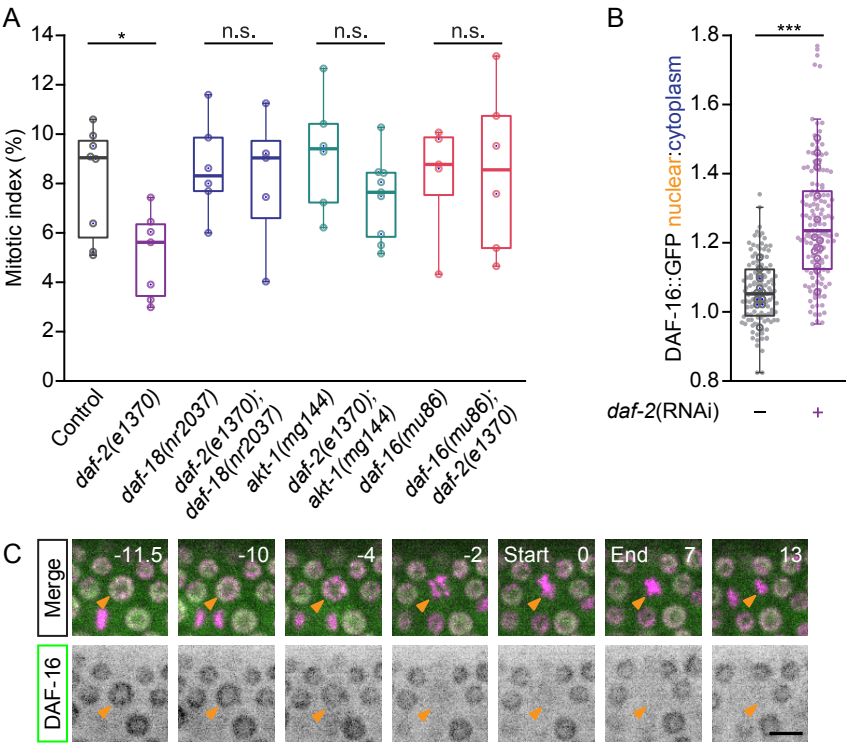

Supplement: S3 Fig — (A) The PZ mitotic index in animals bearing the mutant alleles or allele combinations indicated. daf-18(nr2037), akt-1(mg144) and daf-16(mu86) rescue the lower mitotic index seen in daf-2(e1370) animals. Dots represent one PZ and one PZ was assessed per animal. (B) The DAF-16::GFP nuclear-to-cytoplasmic ratio (N:C) is elevated in GSPCs from animals in which daf-2 was knocked down by RNAi. Small dots represent individual GSPCs, larger dots represent the mean value per gonad arm/PZ. (C) Single time point maximum intensity projections showing DAF-16::GFP nuclear localization over the course of a GSPC mitosis. Numbers indicate time in minutes relative to mitosis start, as inferred from HIS-11::mCH variance. Scale bar = 5 μm. For (A) and (B), boxplots show the median, interquartile range and most extreme values not considered statistical outliers. n.s. = p > 0.05, * = p < 0.05, *** = p < 0.001. Summary statistics and statistical tests used for all figure panels are given in S2 Data. (PDF) [file pgen.1011351.s003.pdf]

S4 Figure

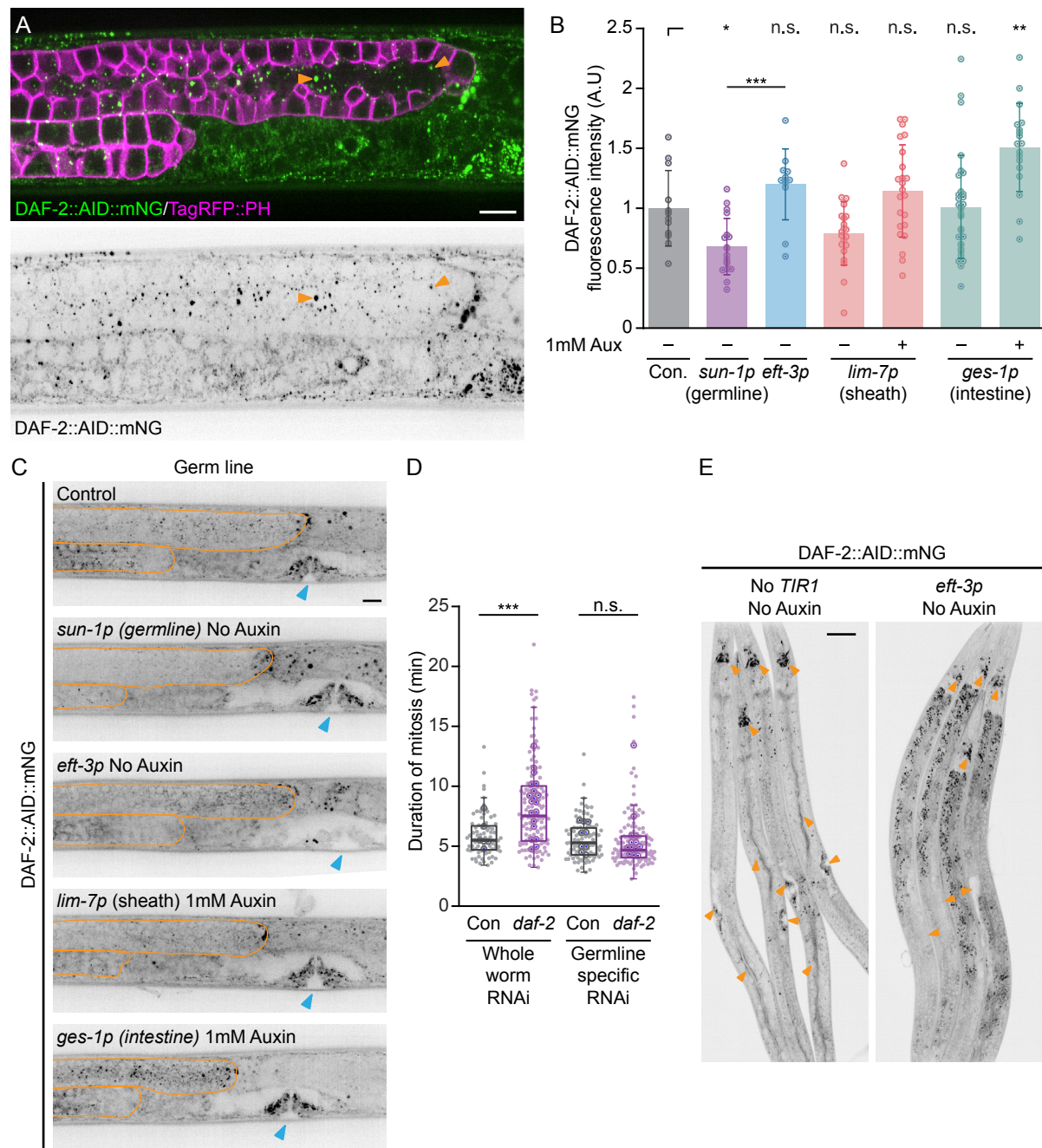

Supplement: S4 Fig — (A) Maximum intensity projection of the distal germ line of a late L4 larvae showing DAF-2::AID::mNG (green) and germ cell membranes (magenta; tagRFP::PH). DAF-2::AID::mNG is shown in inverted grey scale below. DAF-2::AID::mNG in the distal germ line is predominantly found in cytoplasmic puncta (arrow heads) in GSPCs and in the rachis (the shared inner core of cytoplasm to which all germ cells are connected via cytoplasmic bridges). Scale bar = 10 μm. (B) The mean DAF-2::AID::mNG fluorescence intensity per distal germ line, normalized to control animals (no TIR1). Dots represent the mean value per each gonad arm and one gonad arm was assessed per animal. Bar plots show the mean with error bars showing the standard deviation. DAF-2::AID::mNG levels are lower in sun-1p::TIR1 animals without auxin treatment. DAF-2::AID::mNG levels in eft-3p::TIR1 germ lines without auxin treatment are not different from control, but are higher compared to sun-1p::TIR1 germ lines without auxin. Auxin treatment does not deplete DAF-2::AID::mNG in lim-7p::TIR1 or ges-1p::TIR1 germ lines, with slightly elevated levels in ges-1p::TIR1 germ lines following auxin treatment. (C) Maximum intensity projections showing DAF-2::AID::mNG in the germ line (outlined in orange) and developing vulva (blue arrow head) in control animals (no TIR1) and in animals carrying sun-1p::TIR1 and eft-3p::TIR1 without auxin treatment, and in lim-7p::TIR1 and ges-1p::TIR1 with auxin treatment. The vulva is shown as an example of somatic depletion in eft-3p::TIR1. Scale bar = 10 μm. (D) GSPC mitosis is delayed in animals after daf-2 was depleted by RNAi throughout the whole worm but not when daf-2 was depleted in the germ line alone. Small dots represent individual GSPCs, larger dots represent the mean value per gonad arm/PZ. Boxplots show the median, interquartile range and most extreme values not considered statistical outliers. (E) Lower magnification, whole worm images showing partial DAF-2::AID::mNG depletion in [file pgen.1011351.s004.pdf]

S5 Figure

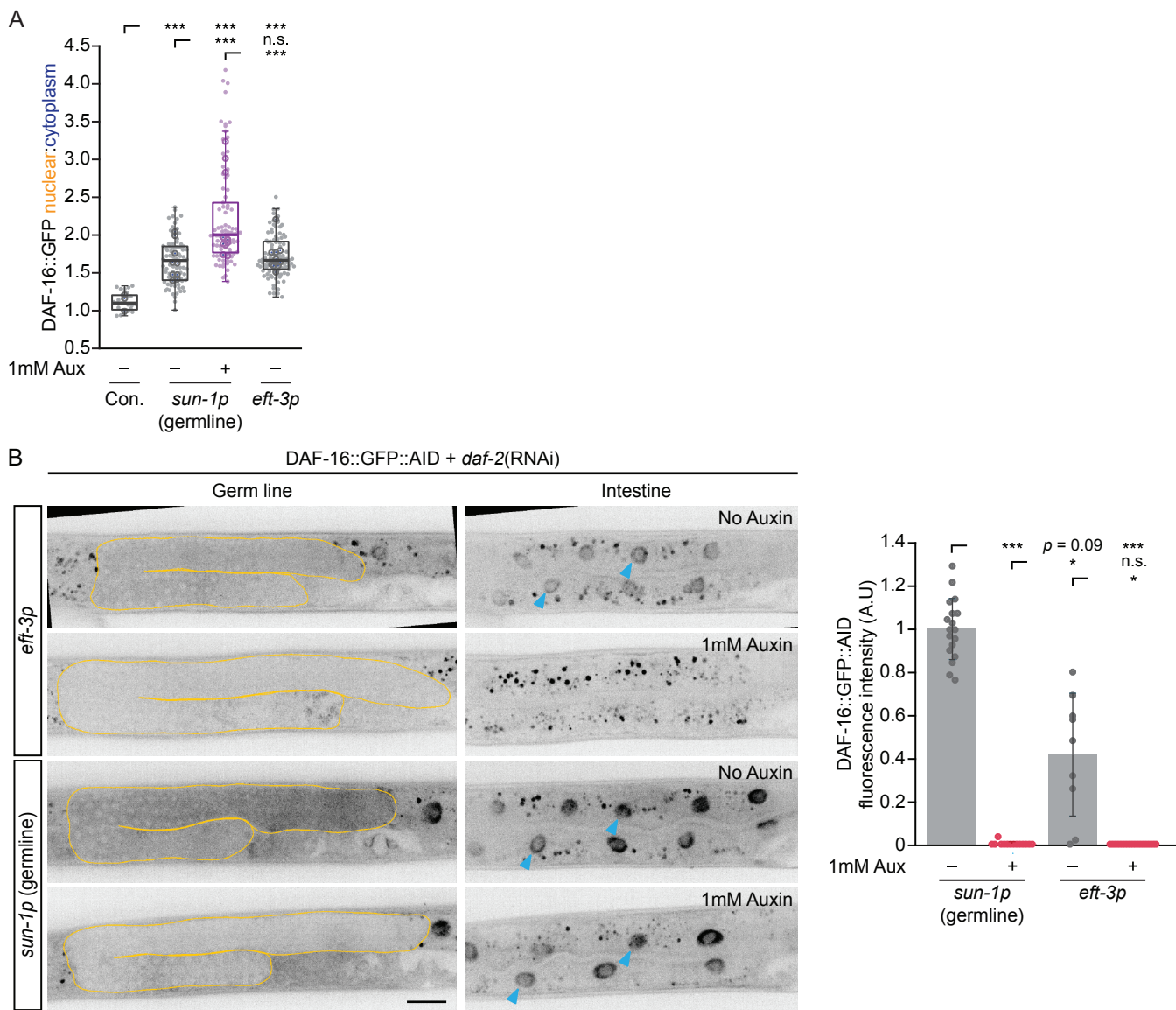

Supplement: S5 Fig — (A) The GSPC DAF-16::GFP nuclear-to-cytoplasmic ratio (N:C) in control animals (no TIR1), sun-1p::TIR1 animals with and without auxin, and eft-3p::TIR1 animals without auxin. The DAF-16::GFP N:C is elevated in sun-1p::TIR1 and eft-3p::TIR1 GSPCs without auxin treatment, compared to control, but increases further in sun-1p::TIR1 animals after auxin treatment. Small dots represent individual GSPCs, larger dots represent the mean value per gonad arm/PZ. Boxplots show the median, interquartile range and most extreme values not considered statistical outliers. (B) Single z-slice sections through the middle of the germ line (orange outline; left), or a basal region through intestinal nuclei (blue arrow heads; right) in the same animals, showing DAF-16::GFP::AID expression in daf-2(RNAi) treated animals. As with DAF-2::AID::mNG, eft-3p::TIR1 leads to partial somatic depletion of DAF-16::GFP::AID without the addition of auxin (compare top intestinal nuclei to those in either of the bottom 2 images). Auxin treatment leads to nearly complete DAF-16::GFP::AID depletion, in both germ line and soma, in eft-3p::TIR1 animals, and robust germ line depletion in sun-1p::TIR1 animals. Scale bar = 20 μm. Quantification of germ line DAF-16::GFP::AID fluorescence for a subset of the animals assayed in Fig 3E is shown on the right. Dots represent the mean value per each gonad arm and one gonad arm was assessed per animal. Bar plots show the mean with error bars showing the standard deviation. For all plots, n.s. = p > 0.05, * = p < 0.05, ** = p <0.01, *** = p < 0.001. Summary statistics and statistical tests used for all figure panels are given in S2 Data. (PDF) [file pgen.1011351.s005.pdf]

**S6 Figure**

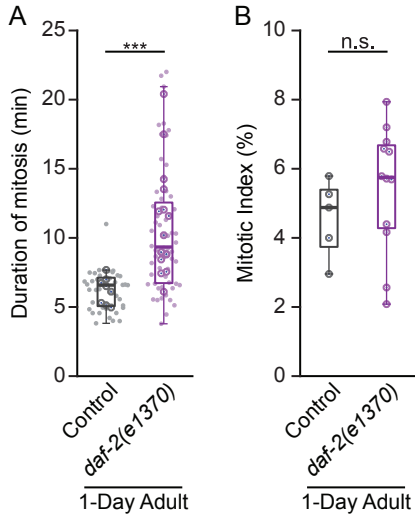

Supplement: S6 Fig — (A-B) The duration of mitosis (A), and the PZ mitotic index (B) of 1-Day adult control and daf-2(e1370) mutant animals. In daf-2(e1370) 1-Day adults, mitosis is delayed but there is no difference in the mitotic index. In (A), small dots represent individual GSPCs, larger dots represent the mean value per gonad arm/PZ. In (B), dots represent one PZ and one PZ was assessed per animal. In both (A) and (B), boxplots show the median, interquartile range and most extreme values not considered statistical outliers. n.s. = p > 0.05, *** = p < 0.001. Summary statistics and statistical tests used for all figure panels are given in S2 Data. (PDF) [file pgen.1011351.s006.pdf]
