# Supplementary material for: Non-autonomous insulin signaling delays mitotic progression in C. elegans germline stem and progenitor cells
Source: PLoS Genet. 2024 Dec 23;20(12):e1011351. doi: 10.1371/journal.pgen.1011351 (PMC11706408; doi:10.1371/journal.pgen.1011351)
Supplement: S1 Table — Unless otherwise indicated, all strains were generated as part of this study, by standard genetic crosses. The source strains for alleles and transgenes are indicated the first time a strain carrying them appears in the table. (DOCX) [file pgen.1011351.s007.docx]

**S1 Table**

| **Strain** | **Genotype** | **Source** |
| --- | --- | --- |
| JDU19 | *ijmSi7 [pJD348/pSW077; mosI_5'mex-5_GFP::tbb-2; mCherry::his-11; cb-unc-119(+)]I; unc-119(ed3)III* | Dr. B. Lacroix |
| ARG50 | *ijmSi7 [pJD348/pSW077; mosI_5'mex-5_GFP::tbb-2; mCherry::his-11; cb-unc-119(+)]I* | This study; JDU19 introgressed into N2 for 5 generations. |
| ARG18 | *ijmSi7 [pJD348/pSW077; mosI_5'mex-5_GFP::tbb-2; mCherry::his-11; cb-unc-119(+)]I; daf-2(e1370)III* | This study; *daf-2(e1370)* from strain CB1370 (CGC). |
| ARG19 | *ijmSi7 [pJD348/pSW077; mosI_5'mex-5_GFP::tbb-2; mCherry::his-11; cb-unc-119(+)]I; daf-18(nr2037)IV* | This study; *daf-18(nr2037)* from strain NR2037 (Dr. J.-C. Labbé). |
| ARG73 | *ijmSi7 [pJD348/pSW077; mosI_5'mex-5_GFP::tbb-2; mCherry::his-11; cb-unc-119(+)]I; glp-1(e2141)III* | This study; *glp-1(e2141)* from strain CB4037 (CGC). |
| ARG101 | *ijmSi7 [pJD348/pSW077; mosI_5'mex-5_GFP::tbb-2; mCherry::his-11; cb-unc-119(+)]I; glp-1(ar202)III* | This study; *glp-1(ar202)* from strain GC833 (CGC). |
| ARG76 | *ijmSi7 [pJD348/pSW077; mosI_5'mex-5_GFP::tbb-2; mCherry::his-11; cb-unc-119(+)]I; mpk-1(ga111), unc-79(e1068)III* | This study; *mpk-1(ga111)* from strain SD939 (CGC). |
| ARG84 | *ijmSi7 [pJD348/pSW077; mosI_5'mex-5_GFP::tbb-2; mCherry::his-11; cb-unc-119(+)]I; let-60(ga89)IV* | This study; *let-60(ga89)* from strain SD551 (CGC). |
| ARG75 | *ijmSi7 [pJD348/pSW077; mosI_5'mex-5_GFP::tbb-2; mCherry::his-11; cb-unc-119(+)]I; daf-7(e1372)III* | This study; *daf-7(e1372)* from strain CB1372 (CGC). |
| ARG74 | *ijmSi7 [pJD348/pSW077; mosI_5'mex-5_GFP::tbb-2; mCherry::his-11; cb-unc-119(+)]I; rsks-1(ok1255)III* | This study; *rsks-1(ok1255)* from strain RB1206 (CGC). |
| ARG106 | *ijmSi7 [pJD348/pSW077; mosI_5'mex-5_GFP::tbb-2; mCherry::his-11; cb-unc-119(+)]I; aak-1(tm1944)III; aak-2(ok524)X* | This study; *aak-1(tm1944)* and *aak-2(ok524)* from strain MR1175 (Dr. R. Roy). |
| ARG22 | *ijmSi7 [pJD348/pSW077; mosI_5'mex-5_GFP::tbb-2; mCherry::his-11; cb-unc-119(+)]I; daf-2(e1370)]III; daf-18(nr2037)IV* | This study |
| ARG46 | *ijmSi7 [pJD348/pSW077; mosI_5'mex-5_GFP::tbb-2; mCherry::his-11; cb-unc-119(+)]I; akt-1(mg144)V* | This study;*akt-1(mg144)* from strain GR1310 (CGC). |
| ARG47 | *ijmSi7 [pJD348/pSW077; mosI_5'mex-5_GFP::tbb-2; mCherry::his-11; cb-unc-119(+)]I; daf-2(e1370)III; akt-1(mg144)V* | This study |
| ARG20 | *ijmSi7 [pJD348/pSW077; mosI_5'mex-5_GFP::tbb-2; mCherry::his-11; cb-unc-119(+)], daf-16(mu86)I* | This study; *daf-16(mu86)* from strain CF1038 (CGC). |
| ARG23 | *ijmSi7 [pJD348/pSW077; mosI_5'mex-5_GFP::tbb-2; mCherry::his-11; cb-unc-119(+)], daf-16(mu86)I; daf-2(e1370)III* | This study |
| ARG2 | *ijmSi31[pJD446_pJD362_Mos2_Pmex-5_mCherry_his11_3'UTRtbb-2]II* | This study; JDU128 (Dr. B. Lacroix) introgressed into N2 for 5 generations. |
| ARG40 | *daf-16(ot971[daf-16::GFP])I; ijmSi31[pJD446_pJD362_Mos2_Pmex-5_mCherry_his11_3'UTRtbb-2]II* | This study; *daf-16(ot971)* from strain OH16024 (CGC)*.* |
| ARG42 | *daf-16(ot971[daf-16::GFP])I; ijmSi31[pJD446_pJD362_Mos2_Pmex-5_mCherry_his11_3'UTRtbb-2]II; daf-2(e1370)III* | This study |
| ARG3 | *ltSi567 [pOD1517/pSW222; Pmex-5::mCherry::tbb-2::tbb-2_3'UTR; cb-unc-119(+)]I* | This study; OD1707 (Dr. A. Desai) introgressed into N2 for 4 generations. |
| ARG61 | *ltSi567 [pOD1517/pSW222; Pmex-5::mCherry::tbb-2::tbb-2_3'UTR; cb-unc-119(+)]I; daf-2(hq363[daf-2::degron::mNeonGreen])III; ieSi38 [sun-1p::TIR1::mRuby::sun-1 3'UTR + Cbr-unc-119(+)]IV* | This study; *daf-2(hq363)* from strain MQD2375 (CGC)  *ieSi38* from strain MQD2375 (CGC). |
| ARG70 | *ltSi567 [pOD1517/pSW222; Pmex-5::mCherry::tbb-2::tbb-2_3'UTR; cb-unc-119(+)]I; ieSi57 [eft-3p::TIR1::mRuby::unc-54 3' UTR + Cbr-unc-119(+)]II; daf-2(hq363[daf-2::degron::mNeonGreen])III* | This study; *ieSi57* from strain MQD2453 (CGC). |
| ARG92 | *daf-16(ot971[daf-16::GFP])I; ijmSi31 [pJD446_pJD362_Mos2_Pmex-5_mCherry_his11_3'UTRtbb-2]II; daf-2(hq363[daf-2::degron::mNeonGreen])III; ieSi38 [sun-1p::TIR1::mRuby::sun-1 3'UTR + Cbr-unc-119(+)]IV* | This study |
| ARG109 | *daf-16(ot971[daf-16::GFP])I; ieSi57 [eft-3p::TIR1::mRuby::unc-54 3' UTR + Cbr-unc-119(+)]II; daf-2(hq363[daf-2::degron::mNeonGreen])III; ltIs37 [pAA64; pie-1::mCherry::HIS-58; unc-119(+)]IV* | This study; *ItIs37* from strain UM226 (Dr. J.-C. Labbé). |
| ARG79 | *ltSi567 [pOD1517/pSW222; Pmex-5::mCherry::tbb-2::tbb-2_3'UTR; cb-unc-119(+)], daf-16(hq389[daf-16::gfp::degron])I; ieSi57 [eft-3p::TIR1::mRuby::unc-54 3' UTR + Cbr-unc-119(+)]II* | This study; *daf-16(hq389)* from strain MQD2433 (CGC). |
| ARG90 | *ltSi567 [pOD1517/pSW222; Pmex-5::mCherry::tbb-2::tbb-2_3'UTR; cb-unc-119(+)], daf-16(hq389[daf-16::gfp::degron])I; ieSi38 [sun-1p::TIR1::mRuby::sun-1 3'UTR + Cbr-unc-119(+)]IV* | This study |
| ARG167 | *ltSi567 [pOD1517/pSW222; Pmex-5::mCherry::tbb-2::tbb-2_3'UTR; cb-unc-119(+)], daf-16(hq389[daf-16::gfp::degron])I; ieSi61 [ges-1p::TIR1::mRUby::unc-54 3' UTR + Cbr-unc-119(+)] II* | This study; *ieSi61* from strain MQD2374 (CGC). |
| ARG87 | *ltSi567 [pOD1517/pSW222; Pmex-5::mCherry::tbb-2::tbb-2_3'UTR; cb-unc-119(+)]I; hqSi11 [lim-7p::TIR1::mRuby::unc-54 3' UTR + Cbr-unc-119(+)]II; daf-2(hq363[daf-2::degron::mNeonGreen])III* | This study; *hqSi11* from strain MQD2383 (CGC). |
| ARG89 | *ltSi567 [pOD1517/pSW222; Pmex-5::mCherry::tbb-2::tbb-2_3'UTR; cb-unc-119(+)]I; hqSi8 [rgef-1p::TIR1::mRuby::unc-54 3'UTR + Cbr-unc-119(+)]II; daf-2(hq363[daf-2::degron::mNeonGreen])III* | This study; *hqSi8* from strain MQD2356 (CGC). |
| ARG113 | *ltSi567 [pOD1517/pSW222; Pmex-5::mCherry::tbb-2::tbb-2_3'UTR; cb-unc-119(+)]I; hqSi10 [myo-3p::TIR1::mRuby::unc-54 3' UTR + Cbr-unc-119(+)]II; daf-2(hq363[daf-2::degron::mNeonGreen])III* | This study; *hqSi10* from strain MQD2379 (CGC). |
| ARG88 | *ltSi567 [pOD1517/pSW222; Pmex-5::mCherry::tbb-2::tbb-2_3'UTR; cb-unc-119(+)]I; ieSi61 [ges-1p::TIR1::mRUby::unc-54 3' UTR + Cbr-unc-119(+)]II; daf-2(hq363[daf-2::degron::mNeonGreen])III* | This study |
| ARG115 | *ltSi567 [pOD1517/pSW222; Pmex-5::mCherry::tbb-2::tbb-2_3'UTR; cb-unc-119(+)]I; hqSi9 [dpy-7p::TIR1::mRuby::unc-54 3'UTR + Cbr-unc-119(+)]II; daf-2(hq363[daf-2::degron::mNeonGreen])III* | This study; *hqSi9* from strain MQD2378 (CGC). |
| ARG107 | *cpSi20 [Pmex-5::TAGRFPT::PH::tbb-2 3'UTR + unc-119 (+)]II; daf-2(hq363[daf-2::degron::mNeonGreen])III* | This study; *cpSI20* from strain LP193 (CGC). |
| ARG45 | *ijmSi7 [pJD348/pSW077; mosI_5'mex-5_GFP::tbb-2; mCherry::his-11; cb-unc-119(+)]I; mkcSi13 [sun-1p::rde-1::sun-1 3'UTR + unc-119(+)]II; rde-1(mkc36)V* | This study; *mkcSi13* and *rde-1(mkc36)* from strain DCL569 (CGC). |
| ARG56 | *ltSi567 [pOD1517/pSW222; Pmex-5::mCherry::tbb-2::tbb-2_3'UTR; cb-unc-119(+)]I; daf-2(hq363[daf-2::degron::mNeonGreen])III* | This study |
| ARG59 | *ltSi567 [pOD1517/pSW222; Pmex-5::mCherry::tbb-2::tbb-2_3'UTR; cb-unc-119(+)]I; ieSi38 [sun-1p::TIR1::mRuby::sun-1 3'UTR + Cbr-unc-119(+)]IV* | This study |
| ARG60 | *ltSi567 [pOD1517/pSW222; Pmex-5::mCherry::tbb-2::tbb-2_3'UTR; cb-unc-119(+)]I; ieSi57 [eft-3p::TIR1::mRuby::unc-54 3' UTR + Cbr-unc-119(+)]II* | This study |
| ARG3 | *ltSi567 [pOD1517/pSW222; Pmex-5::mCherry::tbb-2::tbb-2_3'UTR; cb-unc-119(+)]I* | This study |
| ARG51 | *ltSi567 [pOD1517/pSW222; Pmex-5::mCherry::tbb-2::tbb-2_3'UTR; cb-unc-119(+)]I; daf-2(e1370)III* | This study |
| UM686 | *ltSi567 [pOD1517/pSW222; Pmex-5::mCherry::tbb-2::tbb-2_3'UTR; cb-unc-119(+)]I; mdf-2(lt4::loxP::cb-unc-119(+)::loxP)IV* | This study; *mdf-2(lt4)* from strain OD2174 (CGC). |
| ARG52 | *ltSi567 [pOD1517/pSW222; Pmex-5::mCherry::tbb-2::tbb-2_3'UTR; cb-unc-119(+)]I; daf-2(e1370)III; mdf-2(lt4::loxP::cb-unc-119(+)::loxP)IV* | This study |
| ARG103 | *ijmSi7 [pJD348/pSW077; mosI_5'mex-5_GFP::tbb-2; mCherry::his-11; cb-unc-119(+)]I; mdf-2(lt4::loxP::cb-unc-119(+)::loxP)IV* | This study |
| ARG104 | *ijmSi7 [pJD348/pSW077; mosI_5'mex-5_GFP::tbb-2; mCherry::his-11; cb-unc-119(+)]I; daf-2(e1370)III; mdf-2(lt4::loxP::cb-unc-119(+)::loxP)IV* | This study |
| HR1459 | *bcls39 [lim-7::ced-1::GFP; lin-15(+)]V* | Dr. J.-C. Labbé |
| ARG117 | *daf-2(e1370)III; bcls39 [lim-7::ced-1::GFP; lin-15(+)]V* | This study |
| ARG121 | *mdf-2(lt4::loxP::cb-unc-119(+)::loxP)IV; bcls39 [lim-7::ced-1::GFP; lin-15(+)]V* | This study |
| ARG122 | *daf-2(e1370)III; mdf-2(lt4::loxP::cb-unc-119(+)::loxP)IV; bcls39 [lim-7::ced-1::GFP; lin-15(+)]V* | This study |
| ARG131 | *san-1(ok1580)I; ijmSi31 [pJD446_pJD362_Mos2_Pmex-5_mCherry_his11_3'UTRtbb-2]II; daf-2(e1370)III* | This study; *san-1(ok1580)* from strain RB1391 (CGC). |
| ARG130 | *san-1(ok1580)I; ijmSi31 [pJD446_pJD362_Mos2_Pmex-5_mCherry_his11_3'UTRtbb-2]II* | This study |
| UM225 | *ojIs1 [unc-119(+) pie-1::GFP::tbb-2]V* | (1) |
| UM272 | *eat-2(ad465)II; ojIs1 [unc-119(+) pie-1::GFP::tbb-2]V* | (1) |
| UM362 | *daf-16(mu86)I; eat-2(ad465)II; ojIs1 [unc-119(+) pie-1::GFP::tbb-2]V* | This study |
| UM363 | *eat-2(ad465)II; daf-18(nr2037)IV; ojIs1 [unc-119(+) pie-1::GFP::tbb-2]V* | This study |

1. Gerhold AR, Ryan J, Vallée-Trudeau JN, Dorn JF, Labbé JC, Maddox PS. Investigating the regulation of stem and progenitor cell mitotic progression by in situ imaging. Curr Biol. 2015;25(9):1123-34.
