# Supplementary material for: Non-autonomous insulin signaling delays mitotic progression in C. elegans germline stem and progenitor cells
Source: PLoS Genet. 2024 Dec 23;20(12):e1011351. doi: 10.1371/journal.pgen.1011351 (PMC11706408; doi:10.1371/journal.pgen.1011351)
Supplement: S2 Table — Imaging parameters are listed by experiment type with relevant figures indicated. Imaging parameters were held constant across experiments with occasional exceptions. Where multiple settings were used, the most common are in bold. For spindle pole and mitotic error tracking, variation in imaging parameters was within the range commonly used by our lab and below the threshold for phototoxicity as determined in [24]. For experiments quantifying fluorescence intensity, data were normalized to the same point of reference to permit comparison across experiments. (DOCX) [file pgen.1011351.s008.docx]

**S2 Table**

| **Experiment** | **Microscope** | **Duration (min)** | **Z-slices** | **Z-step (μm)** | **Frame rate (sec)** | **Laser (%)** | | **Exposure (ms)** | | **Figures** |
| --- | --- | --- | --- | --- | --- | --- | --- | --- | --- | --- |
|  |  |  |  |  |  | **488** | **561** | **488** | **561** |  |
| Dual channel imaging of GFP::TBB-2 and HIS-11::mCH (*ijmSi7 [pJD348/pSW077; mosI_5'mex-5_GFP::tbb-2; mCherry::his-11; cb-unc-119(+)]*) | Nikon CSU-X1 | 40 | **27**  **29**  35 | 0.75 | 30 | 2.7  **3**  7 | 3  **5** | 80 | 80 | 1E-F, S1A-B, S4B, 5C-D, S6A-C |
| Dual channel imaging of GFP::TBB-2 and HIS-11::mCH (*ijmSi7 [pJD348/pSW077; mosI_5'mex-5_GFP::tbb-2; mCherry::his-11; cb-unc-119(+)]*) | Quorum Wave FX-X1 | 40 | 26 | 0.75 | 30 | 6 *  **7** * | 10  **15** | 300 ** | | 2B, S3A |
| (* laser wavelength 491; ** dual camera, single exposure) | | | | | | | | | | |
| Dual channel imaging of HIS-11::mCH (*ijmSi31[pJD446_pJD362_Mos2_Pmex-5_mCherry_his11_3'UTRtbb-2]*) and DAF-16::GFP (*daf-16(ot971[daf-16::GFP])*) | Nikon CSU-X1 | 30 | 25 | 0.75 | 30 | 4.8 | 3 | 150 | 80 | 2F-G, S3C |
| Dual channel imaging of HIS-11::mCH (*ijmSi31[pJD446_pJD362_Mos2_Pmex-5_mCherry_his11_3'UTRtbb-2]*) and DAF-16::GFP (*daf-16(ot971[daf-16::GFP])*) | Nikon CSU-X1 | ST | **25**  41 | 0.75 | n/a | **4.8**  5 | 3 | 150 | 80 | 2C-E, 3C, S3B |
| Single channel imaging of mCH::TBB-2 (*ltSi567 [pOD1517/pSW222; Pmex-5::mCherry::tbb-2::tbb-2_3'UTR; cb-unc-119(+)]*) | Nikon CSU-X1 | 40 | **27**  **29**  35  41 | 0.75 | 30 | n/a | 3  **5** | n/a | 80 | 3D-F, S4E-F, 4A, S5C |
| Single channel imaging of GFP::TBB-2 (*ojIs1 [unc-119(+) pie-1::GFP::tbb-2]*) | Nikon CSU-X1 | 40 | **27**  **29**  35  41 | 0.75 | 30 | 5 | n/a | **100**  200 | n/a | 5B |
| Dual channel imaging of GFP::TBB-2 and HIS-11::mCH (*ijmSi7 [pJD348/pSW077; mosI_5'mex-5_GFP::tbb-2; mCherry::his-11; cb-unc-119(+)]*) | Nikon CSU-X1 | 40 | 27 | 0.5 | 15  **30** | 3 | 10 | 100  **200** | 200 | 4C |
| Single channel imaging of HIS-11::mCH (*ijmSi31[pJD446_pJD362_Mos2_Pmex-5_mCherry_his11_3'UTRtbb-2])* | Nikon CSU-X1 | 40 | **37**  **41**  **49** | 0.5 | 30 | n/a | 10 | n/a | **80**  **150** | 5A, S5A |
| Dual channel imaging of mCH::TBB-2 (*ltSi567 [pOD1517/pSW222; Pmex-5::mCherry::tbb-2::tbb-2_3'UTR; cb-unc-119(+)]*) and DAF-2::AID::mNG (*daf-2(hq363[daf-2::degron::mNeonGreen])*) | Nikon CSU-X1 | ST | 27 | 0.75 | n/a | 15 | 3 | 150 | 80 | 3A-B |
| Dual channel imaging of TagRFP::PH (*cpSi20[Pmex-5::TAGRFPT::PH::tbb-2 3'UTR + unc-119 (+)]*) and DAF-2::AID::mNG (*daf-2(hq363[daf-2::degron::mNeonGreen])*) | Nikon CSU-X1 | ST | 61 | 0.5 | n/a | 15 | 3 | 150 | 80 | S4A |
| Dual channel imaging of mCH::TBB-2 (*ltSi567 [pOD1517/pSW222; Pmex-5::mCherry::tbb-2::tbb-2_3'UTR; cb-unc-119(+)]*) and DAF-2::AID::mNG (*daf-2(hq363[daf-2::degron::mNeonGreen])*) | Nikon CSU-X1 | ST | 55 | 0.75 | n/a | 15 | 3 | 150 | 80 | S4C |
| Dual channel imaging of mCH::TBB-2 (*ltSi567 [pOD1517/pSW222; Pmex-5::mCherry::tbb-2::tbb-2_3'UTR; cb-unc-119(+)]*) and DAF-16::GFP::AID (*daf-16(hq389[daf-16::gfp::degron])*) | Nikon CSU-X1 | ST | **35**  **41** | 0.75 | n/a | **7**  **10** | 5 | 150 | 80 | S4D |
| Single channel imaging of CED-1::GFP (*bcls39 [lim-7::ced-1::GFP;lim-15(+)]*) | Nikon CSU-X1 | ST | 69 | 0.75 | n/a | 3 | n/a | 80 | n/a | 4F |

^$^ ST = single timepoint
