# Supplementary material for: Non-autonomous insulin signaling delays mitotic progression in C. elegans germline stem and progenitor cells
Source: PLoS Genet. 2024 Dec 23;20(12):e1011351. doi: 10.1371/journal.pgen.1011351 (PMC11706408; doi:10.1371/journal.pgen.1011351)
Supplement: Attachment — Submitted filename: Cover letter and response to reviews.pdf [file pgen.1011351.s011.pdf]

McGill

Abigail R. Gerhold, PhD
McGill University
Tel: (514) 398-6423
abigail.gerhold@mcgill.ca

Department of Biology
Stewart Biology Building
1205 Dr. Penfield Avenue
Montreal, QC H3A 1B1

PLoS Genetics

October 25th, 2024

Dear Editor,

Thank you for the opportunity to submit a revised version of our manuscript, now entitled "Non-autonomous insulin signaling delays mitotic progression in *C. elegans* germline stem and progenitor cells". We would like to thank the two anonymous reviewers and Dr. Greenstein for their thoughtful reading of our manuscript and their constructive feedback. We were encouraged by the reviewers' positive remarks describing our work as "rigorous" and "carefully done" and by their general level of enthusiasm and interest in our results.

The reviewers raised several important points, which we have made every effort to address. Several major comments related to the biological significance of our results and the relationship between these results and previous work. We have addressed these points with substantial editorial and organizational changes to our manuscript to emphasize both the impact of our work and to clarify how our work is different from prior studies. These changes can be seen in the introduction and first results section, which describes our candidate screen. In addition, we have revised our discussion extensively. In addition, we have added experiments which solidify our conclusion that the insulin pathway is acting cell non-autonomously to influence germline stem and progenitor cell mitosis. We have also included responses to each of the reviewers' individual comments, which motivated several more minor revisions to our manuscript, as detailed below.

Thank you once again for your time and consideration, and we look forward to your response.

Sincerely yours,

Abigail Gerhold
Assistant Professor
Department of Biology
McGill University

Reviewer #1:

1. What is the biological significance of the observations here? Germline proliferation during larval stages is already reduced in daf-2 mutants owing to elongation of the cell cycle, which appears largely to be due to an extension of the G2 phase of the cell cycle (Michaelson et al., 2010). Given that the mitotic cell cycle in the germline has a median length of approximately 2.5 hours in the wild type (Fox et al., 2011), what would be the impact of the extensions of M phase observed here? Addressing this point would help general readers better appreciate the biological relevance of this study.

Response: Our goal with this work was to use GSPCs to identify interactions between signaling pathways and events during mitosis relevant for proper chromosome segregation. As in many other species/cell types, mitosis in GSPCs occupies a relatively small fraction of the overall cell cycle duration – the GSPC cell cycle (G1-S-G2-M) lasts ~6-8 hours (ref 12, Hubbard and Schedl, 2019, and refs therein) and mitosis (NEBD to anaphase) is < 6 minutes, on average. Therefore, the magnitude of even a 2-fold mitotic delay will be on the order of minutes and, correspondingly, make a relatively minor contribution to the overall duration of the cell cycle. However, these delays are significant from the perspective of mitosis itself, as they indicate that cells either have defects in spindle assembly and are thus taking longer to satisfy the spindle assembly checkpoint (as is the case for caloric restriction) or they have other changes that delay the metaphase-to-anaphase transition (e.g. delays in checkpoint silencing, as we speculate is the case for *daf-2* mutants).

In hindsight, and in light of this comment and those of Reviewers 2 and 3, we recognize that we failed to clearly communicate the main motivation for and significance of our work and how our work relates to previous studies, which have focused on cell cycle regulation in GSPCs. We have attempted to remedy this shortcoming with the following changes:

1. We revised our Introduction to emphasize mitotic regulation by signaling pathways and to better differentiate our work/main question from previous studies in GPSCs, which have focused on GSPC proliferation/cell cycle regulation.

2. We restructured the first Results section (*A candidate screen for signaling pathways that affect the duration of mitosis uncovers daf-2/IGFR*) so that we start by describing the motivation for our candidate screen and our approach, before introducing the signaling pathways we targeted. We added a schematic to Fig. 1 to illustrate this. We also emphasized that our goal in assessing the number of PZ cells and the GSPC mitotic index was to validate the strains that we generated before performing live imaging.

3. We removed one paragraph from the Results section *Reduced IIS delays mitosis via the spindle assembly checkpoint but does not compromise mitotic fidelity*, which discussed the genetic interaction between *daf-2* and the checkpoint gene *mdf-2* with regards to sterility. We had speculated that this interaction related to cell cycle regulation rather than mitotic

regulation. We decided that this observation was tangential to our main question and distracting.

4. We removed all reference to cell cycle regulation from the final Results section (*Reduced IIS and caloric restriction delay mitosis via different pathways*). In this section, we test the genetic requirements for mitotic delays following caloric restriction. Whether these genes/pathways also mediate the effect of caloric restriction on GSPC cell cycle progression, as assessed by changes in the mitotic index, is not directly relevant to our main question and distracted from our focus on mitosis.

5. We reorganized and revised the Discussion substantially to better explain the biological significance of the delays we have observed in *daf-2(rf)* mutants and other conditions. The first paragraph now reads:

   > Here we have investigated the signaling requirements for proper GSPC mitosis as a model for how mitosis in stem and progenitor cell is regulated in vivo, in the whole-animal context. Through our candidate screen we found that reducing the activity of daf-2/IGFR, daf-7/TGF-β or glp-1/Notch prolongs the duration of mitosis in GSPCs. As only a subset of the mutant backgrounds in our screen delayed GSPC mitosis, we think it unlikely that these effects are generic response to perturbed signaling compromising animal health. Our data also suggest that delayed cells from different mutant backgrounds exhibit different combinations of spindle features, which may indicate specificity in terms of the mitotic processes that are affected. This idea is further supported by our results showing that mitotic delays in daf-2/IGFR mutants and in animals subjected to caloric restriction can be suppressed genetically, but rely on different downstream signaling effectors and are associated with different phenotypes in terms of mitotic fidelity. Finally, while the absolute magnitude of these delays is small (on the order of minutes), they are significant given the normal duration of GSPC mitosis (< 6 min, on average), and similar in magnitude to those seen following targeted perturbation of kinetochore-microtubule attachments (22). Together, these results argue that the changes we have observed in the duration of GSPC mitosis are likely the product of specific interactions between signaling pathway effectors and mitotic events. Whether these changes are adaptive (e.g. increasing checkpoint activity to buffer cells from environmental fluctuations) or indicative of mitotic vulnerabilities (e.g. changes in gene expression or protein activity that compromise spindle assembly) requires additional investigation.

   We then focus the rest of the discussion on two points: (1) the possible causes of GSPC mitotic delays when IIS is reduced and (2) the signaling mechanism by which this information is transduced.

2. A concern is that that physiology of daf-2 mutants makes them more sensitive to the conditions used for the time-lapse recordings (e.g., absence of food, compression, phototoxicity). Given that the mitotic index of daf-2 mutants was not reported to be affected in the adult stage (Michaelson et al., 2010), I wonder whether the authors would observe similar extensions of M phase in the adult stage. If not, this would a good control and might provide an additional contrast to the prior work. Further, in their Discussion section, the authors speculate

on potential mechanisms. On the face of it, the mechanisms they envision and discuss would not seem to be limited exclusively to larval stages.

Response: We agree with the reviewer that this could be a concern, and it is difficult to exclude completely, since there is no alternative methodology by which the same information could be obtained under difficult conditions. However, we do not think that it is a major confounding factor here for the following reasons:

1. *daf-2* mutants are resistant to multiple stressors and most of this resistance is mediated by *daf-16* (see ref 52, Murphy and Hu, 2013, section 3.3, and refs therein). While the stressors are not identical, we might expect *daf-2* mutants to be less sensitive to stresses induced by our imaging conditions, while *daf-16* mutants should be more sensitive. However, mitosis is delayed in *daf-2* mutants and no different from controls in *daf-16* mutants. Based on this, and the lack of mitotic delays in other stress-sensitive genetic backgrounds (e.g. *aak-1/aak-2(0)*, 71 Apfeld *et al*. 2004), we think that the mitotic delays we have reported in *daf-2* mutants, and for other mutations, are more likely to reflect physiologically relevant interactions between the mutation/signaling pathway and mitotic events.

2. We have previously determined the range of imaging conditions (e.g. levamisole concentration, compression, laser intensity) that nominally wild-type worms will tolerate before we start to see mitotic phenotypes, like delays, in GSPCs (Zellag et al. 2021, ref 24). The conditions that we use routinely are well below the threshold at which we start to see an effect in wild-type animals. While this does not exclude the possibility that mutant strains are more sensitive to the stresses associated with live-cell imaging, it does suggest that our conditions are minimally stressful.

3. We have shown that the primary factor affecting GSPCs during live-cell imaging is removal from food, which leads to fewer GSPCs entering mitosis and a slight increase in the duration of mitosis in those cells that do divide (Zellag et al. 2021, ref 24). In an effort to directly address the reviewer's comment, we assessed this for our original *daf-2* and control data from the candidate screen (40 min movies) and in a new set of longer movies (90 min) for *daf-2* mutants and controls. Our idea was that if *daf-2* mutants were more sensitive to our imaging conditions, we should see a more rapid decrease in mitotic entries and a more rapid increase in the duration of mitosis in *daf-2* mutants over the course of imaging. We were encouraged to see that *daf-2* mutants were not significantly different from controls, suggesting that they are no more sensitive to our imaging. We include this data below. In all panels, purple data is from *daf-2(e1370)* animals and grey data is from control.

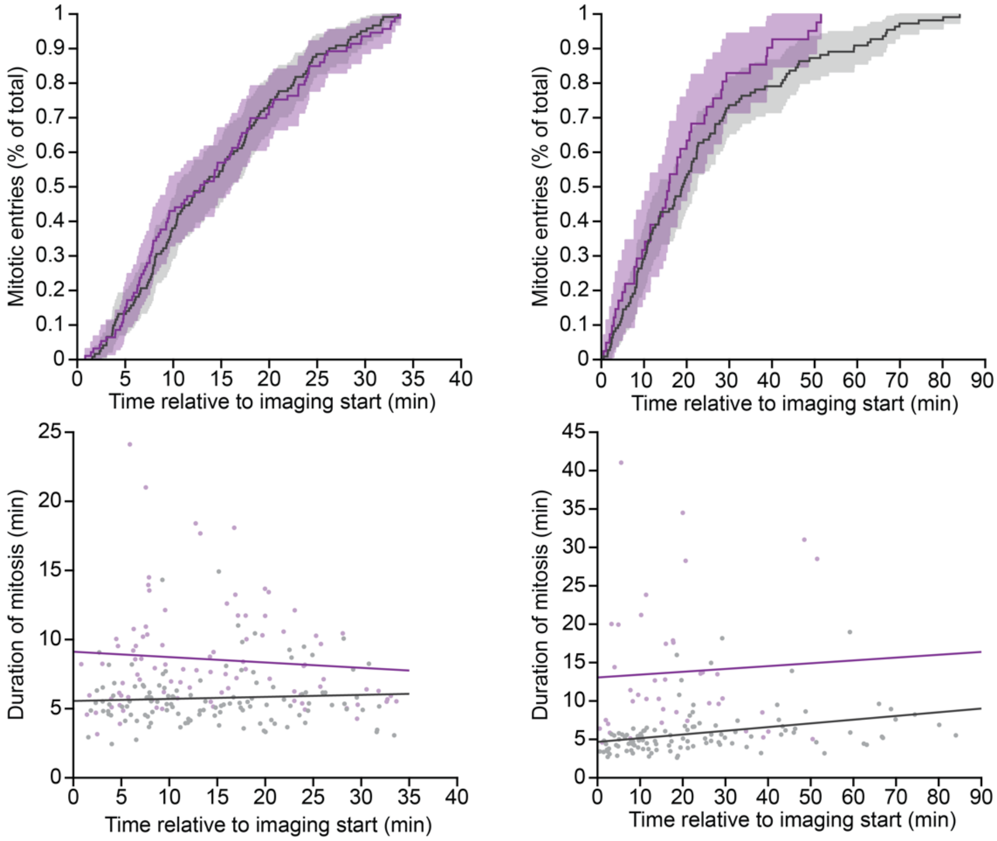

We also addressed the question of whether *daf-2* mutants would show mitotic delays in adults. We found that the GSPC mitotic index was not decreased in 1-day *daf-2(e1370)* adults, in line with previous reports; however, GSPC mitosis was delayed, as in L4 larvae. We think that this result is interesting since, as the reviewer indicates, it suggests an additional difference between how *daf-2* interacts with the GSPC cell cycle versus mitosis. The reviewer is also correct that we might expect this result based on the mechanisms we propose for how *daf-2*/insulin signaling could affect mitosis – these should not be limited to larval GSPCs. We also note that caloric restriction delays GSPC mitosis in adults (Gerhold et al. 2015, ref 22). We reference these results in our revised discussion and have included this data in S6 Fig.

3. I'm not sure it is fair to say here that DAF-2 insulin signaling regulates germline stem/progenitor cells (e.g., in the Title and Abstract). Rather, the results simply indicate that germline stem/progenitor cells are affected in the mutant backgrounds in that the duration of M phase is extended.

Response: We have revised the manuscript, including Title and Abstract, to avoid implying that we have established a direct regulatory relationship between insulin signalling and GSPC mitosis. We have replaced most instances of "regulate" with more direct and/or precise language. The title now reads: "Non-autonomous insulin signaling delays mitotic progression in *C. elegans* germline stem and progenitor cells"

4. Table S1. Strains carrying bcIs39 were generated with the lin-15(+) marker (not lim-15).

Response: Fixed. Thank you.

Reviewer #2:

1) The significant weakness of the manuscript is that the biological significance of the finding reported here for regulation of the mitotic cell cycle in the C. elegans stem-progenitor zone is unclear/not well explained. The current manuscript thus misleads the readership of PLoS Genetics into thinking that this work explains known germline development/physiological effects and signaling gene mutant phenotypes. The unaddressed question is – does the observed mitotic delays in the reported condition explain the germline biology that is observed in these conditions, as implied by Figure 1B and text. The authors must cover these issues in the Discussion, at least.

Published work of others showed that the cell cycle time (S phase, G2 and M) in wild type young adults is ~6.5 hr. The cell cycle time in late larval development is unknown and is thought to be faster than in adults, but is clearly multiple hours long (liberal estimate, 4 hrs). In this study, samples were examined in late L4 where the biology is that this time is nearing the end of the larval expansion of the PZ to yield the young adult PZ size. Changes in cell cycle time need to be considered and explained in the context of the germline biology and signaling that at least some of the readership of PLoS Genetics will know.

Cheng et al. rigorously show that in late L4, during larval expansion of the PZ, the duration of mitosis in daf-2(rf) is increased by a median of 2.2 min (Fig 1F) or 4.4 min per cell (Fig 2B) [average median increase of 3.3 min per cell].

(A) Published work of others showed that daf-2(rf) has fewer cells in the young adult PZ and has a reduced mitotic index. Time course studies show that there are fewer PZ cells in the adult (~130 cells in daf-2(rf)) compared to ~220 cells in wild type) is a result of reduce expansion of the PZ pool from early L3 to young adult (Michaelson et al., 2009).

The biological question of interest is - does the increase in duration of mitosis fully explain the defect in larval expansion in daf-2(rf)? The manuscript, as written, implies that it does, but actually does not address this central point, which was provided as a motivation for the study (Fig 1B, text, citations). If we assume an L4 cell cycle time of 4 hours, an average 3.3 min median mitotic phase delay represents only a 1.4% increase in cell cycle length. Will a 1.4% increase in cell cycle length result in the production of 125 PZ cells in daf-2(rf) versus 220 PZ cells in wt during expansion from ~40 cells in early L3 to young adult (~20hrs)? This reviewer don't know, but it opens the strong possibility that the increase in duration of mitosis does not explain or explains only part of the effect on daf-2 signaling caused by the e1370 mutation.

B) Cheng et al. rigorously show that in late L4 the duration of mitosis increased in time by a median of 1.7 min per cell in glp-1(rf) (Fig 1F). The audience of this manuscript may go away with the idea, even though not directly discussed, that the increase in duration of mitosis during larval development (asses in late L4) leads to the smaller PZ in young adult glp-1(rf). It is reasonable to assume that the cell cycle time of glp-1(rf) at 20oC is 6.5 hrs, given that wt and glp-1(rf) have the same length at 15oC (~9 hrs, Fox and Schedl, 2015). A 1.7 min delay in mitosis corresponds to 0.4% of the cell cycle. It seems unlikely that a 0.4% increase in cell cycle length explains the decreased number of PZ cells in young adult glp-1(rf). Instead, the more likely explanation is that it is the decreased number of stem cells in glp-1(rf), based on published work using staining for stem cell markers, and experiments showing that increasing the number of stem cells correspondingly increases the size of the PZ.

C) What aspect of germline biology during caloric restriction does the mitotic delay explain?

D) This raises the possibility that the observed effect on mitotic phase duration, in at least some of the genetic backgrounds/diet tested, is to counteract conditions that increase the frequency of mitotic chromosome segregation defects (possible biology explained), rather than to change cell cycle length and thus PZ dynamics as part of a process to modify timing and number of self/mated progeny produced.

Response: By our reading, the comments expressed here by Reviewer 2 (point 1 A-D, above) are interconnected and related to point 1 from Reviewer 1. As noted in our response to Reviewer 1, we recognize that we did not adequately communicate the main motivation for our work. As a result, we also did not succeed in drawing a distinction between our work and the aspects of GSPC biology that have been most heavily studied – cell cycle regulation and regulation of the mitosis/meiosis decision. As described in our response to Reviewer 1, point 1, we have revised our manuscript to address this issue. We refer Reviewer 2 to our response above for a list of the changes that we have made. Below we respond to Reviewer 2's comments directly.

We do not think that the mitotic delays that we have observed in some mutant backgrounds, and following caloric restriction, make a meaningful contribution to changes in the overall duration of the cell cycle and/or the number of GSPCs under these conditions. Mitotic delays are on the order of minutes, while the cell cycle is hours long. We also do not think that the

mitotic delays we observed in *glp-1* mutants during our candidate screen contribute to the loss of GSPCs in this background. Fewer GSPCs in *glp-1* mutants has been attributed to premature differentiation (Austin and Kimble, 1987, ref 31 and Fox et al. 2015, ref 14) and we do not think our results change this conclusion. It was not our intention to imply such a connection for any of the mutants that we tested, and we hope that this is clearer in our revised manuscript.

Instead, we believe that the changes to mitotic duration that we observe in some mutant backgrounds and following caloric restriction are due to changes in spindle assembly and/or spindle assembly checkpoint activity. In cases where these changes increase the frequency of chromosome segregation (e.g. caloric restriction), they are likely to reflect signaling and/or physiological environments that compromise mitotic fidelity and/or increase the requirement for a robust spindle assembly checkpoint. In cases where the frequency of chromosome segregation errors does not increase (e.g. *daf-2(e1370)*), as the reviewer suggests in comment 1D and as we had proposed in our Discussion, they are more likely to reflect interactions with the spindle assembly checkpoint itself and may serve to protect cells under conditions that might otherwise compromise mitotic fidelity. We have also attempted to make these points more evident in our revised manuscript.

In general, our goal was to use GSPCs to identify interactions between signaling pathways and events during mitosis that are important for proper chromosome segregation (i.e. mitotic fidelity). While cell cycle regulation and the mitosis/meiosis decision have been studied extensively in GSPCs, we know relatively little about how changes in signaling and/or animal physiology affect mitotic fidelity in these cells. We targeted signaling pathways that were already known to influence other aspects of GSPC biology and that regulate cell proliferation and/or differentiation in many species. However, because our approach focuses on GSPC mitosis, we hoped to uncover new roles for these pathways, which might be relevant for mitotic cells *in vivo* more generally (e.g. stem and progenitor cells in other species/contexts). For this reason, we focused our manuscript on the implications of our work for mitosis/mitotic fidelity rather than germline biology specifically.

E) Published work has shown that signaling and regulation of stem and progenitor cells during larval development, in the adult, and in the distinct diapauses can show significant differences. Therefore, the authors need to make it clear in the Abstract, Introduction, Results and the Discussion that this working is examining signaling during larval expansion of the PZ, at late L4. Furthermore, when describing different genes and signaling pathways, the authors should indicate in what stage(s) these studies were conducted.

Response: In re-organizing the first section of our Results to make the motivation for our candidate screen and our approach clearer/more prominent, the following sentence is now part of the first paragraph, which we hope avoids any ambiguity regarding the stage of development that we have focused on.

> We focused our analysis on the L4 larval stage of development (Fig 1A), as GSPCs proliferate extensively during this time and may be more sensitive to changes in environmental and/or developmental signaling (3, 29, 30).

We have also made minor revisions to the paragraph describing the genes/signaling pathways we tested in our screen to add developmental stage(s), where this information is relevant (i.e. the role of pathway/gene may be specific to a given stage/context) and was not already present.

In revising our Introduction, we now introduce the idea of larval GSPC proliferation, which builds the germ line during development, and adult GSPC proliferation, which sustains gamete production.

Finally, in response to Reviewer 1, point 2, we tested whether mitosis was also delayed in GSPCs in *daf-2(e1370)* adults and found that it is. The delays generated by caloric restriction are also seen in adults (Gerhold et al., 2015, ref 22). We reference this result in the Discussion as a difference between the way insulin signaling affects GSPC proliferation versus mitosis.

Minor points:

2) The abbreviation NEBD is not defined on line 147.

Fixed. Thank you.

3) It would be useful to discuss the specific mitotic features that seem to be important in the different genotypes in the paragraph starting on line 156 as the current description is vague.

We now refer to these as “spindle features” to be more precise and define them in the first paragraph of our revised Results section as “spindle length and dynamics throughout mitosis”. We have also added these sentences to the final paragraph:

> We found that strains with mitotic delays were unalike in both the magnitude and directionality of change for most spindle features (S2B Fig). For example, fluctuations in spindle length during spindle assembly were reduced in *daf-2(rf)*, increased in *glp-1(e2141)* and unchanged in *daf-7(e1320)*, relative to controls (S2B Fig).

4) In Figure 3 the authors use strains containing DAF-2::AID::mNG, TIR1::Ruby, mCH::TBB-2, and DAF-16::GFP. How are the two red and two green fluorescent proteins separated in these experiments?

mCH::TBB-2, HIS-11::mCH and HIS-58::mCH are all significantly brighter than the TIR1::mRuby such that with our live-cell imaging settings, tracking and pairing spindle poles and/or tracking nuclei is not affected. We do not quantify fluorescence, so any additional background from the mRuby is not an issue here.

For the experiment where we measured DAF-16::GFP while depleting DAF-2::AID::mNG in either the germ line or soma: the DAF-16::GFP signal is significantly brighter than DAF-2::AID::mNG in the germ line, and, with the conditions we used to detect DAF-16::GFP, the germline DAF-2::AID::mNG fluorescence intensity (FI) is close to or below our detection threshold. However, we fully agree with the reviewer that we should have accounted for this directly and thank them for bringing it to our attention. We have repeated this experiment, controlling for DAF-2::AID::mNG FI directly, as described in the revised Methods:

> For data shown in S5A Fig., where DAF-16::GFP was measured in animals carrying DAF-2::mNG::AID, an additional background correction was applied by measuring, in parallel, the germline FI of DAF-2::mNG::AID in animals carrying the same TIR1 drivers, but without DAF-16::GFP, as described below. These values were subtracted from the relevant DAF-16::GFP measurements (e.g. *sun1p::TIR1* DAF-2::mNG::AID 1mM Aux from *sun1p::TIR1* DAF-2::mNG::AID DAF-16::GFP 1mM Aux) prior to calculating the nuclear-to-cytoplasmic ratio.

While the overall result does not change, in repeating this experiment we were also able to include an additional control which now allows us to discuss this experiment as follows:

> To determine whether DAF-16/FoxO also acts cell non-autonomously, we first assessed whether germline (*sun1p::TIR1*, with and without auxin) and/or partial somatic (*eft3p::TIR1*, no auxin) depletion of DAF-2/IGFR affected the localization of DAF-16::GFP in GSPCs. We found that DAF-16::GFP was enriched in GSPC nuclei in all three conditions relative to control, but was highest when DAF-2/IGFR was depleted from the germ line by auxin treatment (S5A Fig), consistent with a germline-autonomous role for DAF-16/FoxO. However, since GPSC mitosis is not delayed when DAF-2/IGFR is depleted from the germline, yet under these conditions nuclear DAF-16::GFP is at its highest, these results further suggest that nuclear localization of DAF-16/FoxO in GSPCs does not play a major role in determining the duration of GSPC mitosis.

5) Line 203. Having 25 min is gratuitous, given that it is clearly an outlier in the data. It is well known the live imaging of the distal germline without inducing arrest or other aberrant behaviors is challenging; significant time delayed outliers may arise from such live distal germline imaging issues.

We respectfully disagree. We have optimized our live-cell imaging conditions to minimize stresses associated with mounting and/or imaging and this gives us confidence that when strongly delayed cells are observed, they likely represent the true range of the phenotype (Zellag et al., 2021 and 2022, ref 24 and ref 23). Given that we see cells with +/- 25 min durations of mitosis in *daf-2* mutants across 3 independent experiments, and not in any of our control strains (Fig 1F, 2B and 4A), we do not think it is unreasonable to state this value as the upper limit of the *daf-2* phenotype.

6) In Figure 3D the authors indicate that germline degradation of DAF-2 has no effect on mitosis but there appears to be a statistically significant reduction in the duration from the figure. The authors should state this result in the text and at least speculate why they would observe this

effect, or is it that while the result is statistically significant it is so small as not to be biologically significant?

In the Results and the Legend for Fig 3D, we described this result as "germline DAF-2/IGFR depletion did not delay GSPC mitosis" (Results) and "Auxin treatment of *sun-1p::TIR1* animals depletes DAF-2::AID::mNG in the germ line … but does not delay GSPC mitosis." (Legend). The reviewer is correct that the duration of mitosis in these animals is shorter than controls; however, the difference is small, and may be an instance of statistical rather than biological significance. We have revised our description of this experiment in Results to read:

> Surprisingly, germline DAF-2/IGFR depletion did not delay GSPC mitosis; rather, we observed a marginal, but statistically significant, decrease in the duration of mitosis.

7) In line 251 the authors state they cannot fully exclude the possibility the germline autonomous DAF-16 regulates mitotic timing, but they have provided no information in support of the idea. The authors should remove the statement or explain why this cannot be excluded.

Because DAF-16::AID was depleted in both the germline and soma when we used *eft-3p::TIR1*, our results did not let us conclude that depletion of DAF-16 in the soma is sufficient to block mitotic delays in GSPCs. However, we now include additional experiments where we have depleted DAF-16 in the intestine. This is sufficient to rescue *daf-2*(RNAi) GSPC mitotic delays, and we find no evidence for DAF-16 depletion in the germline (Fig 3F-G). We have revised this section of the Results to incorporate this new data and, in so doing, have removed this statement.

8) In line 261 the authors state that the intestine and gonadal sheath are the primary sites of DAF-2 activity, but could other cell types also be at play that were not tested? The authors should edit this statement to not exclude the possibility that DAF-2 may also function elsewhere.

We have revised this sentence to now read:

> Taken together, our data strongly suggest that DAF-2/IGFR acts cell non-autonomously to affect mitotic timing in GSPCs, and that, of the tissues tested, the intestine and gonadal sheath cells are the principal foci of DAF-2/IGFR activity.

This idea is also now discussed in our revised Discussion.

9) In lines 280 and 282 the authors describe comparisons between genotypes as "the same" when formally they are failing to reject the null hypothesis of equivalent frequencies. These descriptions should be changed to describe the comparisons as not significantly different.

We have revised this section to now read:

> The frequency of mitotic errors was low in both control and *daf-2(rf)* GSPCs, suggesting that if spindle assembly is compromised in *daf-2(rf)* cells, checkpoint-dependent mitotic delays are sufficient to ensure these cells achieve biorientation prior to anaphase onset. In *mdf-2(0)* GSPCs we observed significantly more mitotic errors, as would be expected for checkpoint-null cells, but surprisingly, the frequency of mitotic errors was not significantly different between these cells and *daf-2(rf); mdf-2(0)* double mutants.

10) In the paragraph starting on line 292, the relationship between the amount of mitotic germ cells and sterility is unclear. Could these be two entirely independent phenotypes? In Figure S5B,C the number of mitotic cells is reduced in daf-2 mutants but sterility is unchanged, suggesting they are unrelated phenotypes. Can the authors provide direct evidence that the amount of mitotic germ cells is necessary for fertility?

In an effort to focus our manuscript on mitosis, as opposed to cell cycle regulation, we have removed this section. In response to Reviewer 2's comment – here we were speculating as to the cause of the genetic interaction that we observed between *mdf-2(0)* and *daf-2(e1370)*. We were using the number of mitotic cells as an indication of a defect in cell cycle progression, which has been shown for for *daf-2(e1370)* (Michaelson, 2010 ref 29) and for *mdf-2(0)* (Lara-Gonzalez *et al.* 2019, ref 63), and which we thought, in combination, might make worms more likely to develop into sterile adults, as is seen when more severe cell cycle perturbations are imposed (e.g. *cdk-1(RNAi)*).

11) It would be good to go into more detail about which spindle features relate to which genotypes in the paragraph on line 352.

We have added more details to this point to the Results section, in response to point 3 above. We have expanded the Discussion to address other major points and, in the interests of overall length, prefer to keep this section as is.

12) On line 408 the authors indicate that germline depletion of DAF-2 had "no effect" on the number of mitotic cells but it is more accurate to say there was no significant difference.

We have removed all reference to this data in response to Reviewer 3 comment 3.

13) In line 666 GSPCs are described as GSCs.

Fixed. Thank you.

14) All figure panel where quantitative data is shown need to have more information on the statistical tests, description of the graphical features, and n values for the groups in the legend.

To keep our legends concise, we had described our data plots in the Method section (Statistics and figure assembly) and provided all summary statistic in S2 Data - Summary statistics for all

figures, including ‘n’, *p* values, descriptive statistics (e.g. median, quartiles, etc.) and the statistical tests used, with the null hypothesis being tested. We had forgotten to reference S2 Data in all our figure legends and thank the reviewer for bringing this to our attention. We have added plot descriptions and included a reference to S2 data for all our figure legends.

15) In Figure 1B the worm gene name for TOR is missing.

Fixed. Thank you.

16) Figure 2D needs a genotype label.

Added.

17) Plots showing DAF-16::GFP nuclear:cytoplasm should start at 0 on the y axis.

This compresses the data and as a result masks some of the variance. For this reason we prefer to use y-axes that display the full range of the data and not more.

18) Figure 4C,D should show p-values between all the groups.

For D, we only compared *daf-2(e1370)*; *mdf-2(0)* with *mdf-2(0)*. For C, we have added stats (i.e. n.s., ***, etc.) to the figure panel for the comparisons that we describe in the Results. The p values are in S2 data.

19) Figure 4F may be better represented as a stacked bar graph showing the proportion of gonads binned into the number of apoptotic bodies.

Thank you for the suggestion. We tried this (see below), but decided that representing the data with a beeswarm + box plot was preferable so that each gonad is visible as a data point.

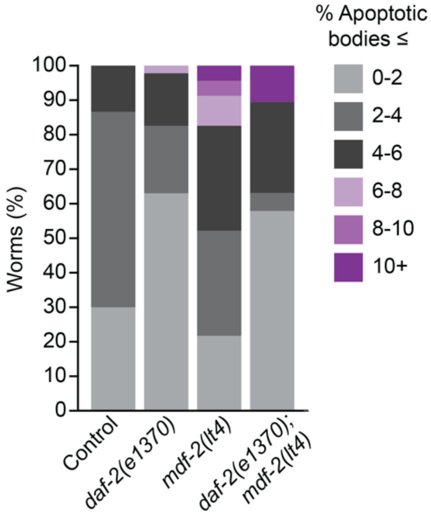

20) In Figure S2D, the points should be labeled as delayed mitotic cells.

Added.

21) An arrow should be added to Figure S3C to point out which nucleus to focus on.

Added.

22) The arrows in Figure S4D are not pointing at the intestinal nuclei.

Fixed.

Reviewer #3:

Major points:
1. The authors employ AID with eft-3 and lim-7::TIR1 strains. How was the eft-3 strain validated to ensure knock down only in somatic tissues with no effects on the germ line? Along the same lines, to my knowledge the lim-7::TIR1 has not been validated to make sure that only sheath cells show AID degradation. This has to be rigorously addressed to validate the provocative results.

Response: The eft-3p::TIR1 strain was generated in Zhang et al. (2015) (ref 60) where they report that they do not see red fluorescence (TIR is tagged with mRuby in these strains) in the germ line. However, other groups have referred to this same eft-3p::TIR1 insertion as driving TIR1 in “all cells” (Zhang et al. 2022, ref 61). For our experiments, we assessed this directly. We measured the fluorescence intensity (FI) of the protein that we were targeting for depletion (DAF-2::AID::mNG or DAF-16::GFP::AID) in the germ line and visually assessed different somatic tissues (vulva, intestine and head neurons, depending on the experiment).

In eft-3p::TIR1 DAF-2::AID::mNG no auxin worms, germline DAF-2 FI was higher than in our sun-1p::TIR1 DAF-2::AID::mNG no auxin worms (Fig 3 A-B and S4B-C Fig). The fact that we see mitotic delays in the eft-3p::TIR DAF-2::AID::mNG worms, while mitosis is not delayed in the sun-1p::TIR1 background, with or without auxin (DAF-2 lower or undetectable in the germ line, respectively), led us to conclude that depleting DAF-2 in the germ line is not sufficient to delay GSPC mitosis, but partial DAF-2 depletion in the soma is.

In eft-3p::TIR1 DAF-16::GFP::AID worms, we saw that, without auxin, DAF-16::GFP::AID FI in the germline was lower than in sun-1p::TIR1 DAF-16::GFP::AID no auxin worms, and after auxin treatment, DAF-16::GFP::AID was ~undetectable in the germ line in both strains (Fig S5B). Because DAF-16 was depleted in both the soma and germline in eft-3p::TIR1 DAF-16::GFP::AID worms, following auxin treatment, we indicated that we were not able to fully exclude the possibility that DAF-16 was also functioning in the germ line to delay mitosis.

In our revised manuscript, we include new data showing that DAF-16 depletion in the intestine is sufficient to suppress *daf-2(RNAi)*-induced mitotic delays in GSPCs. We have included this new data in Fig 3F-H. Based on our original results and this new data, we conclude that germline DAF-16 is not responsible for GSPC mitotic delays when *daf-2* activity is reduced. Instead, DAF-16 activity in the soma is responsible, with the intestine playing a major role.

The lim-7p::TIR1 strain was reported by Zhang et al. (2022) (ref 61). They characterized the expression pattern of the lim-7 promoter using mCherry::NLS such that expressing cells will have red nuclei and showed images to suggest that lim-7p::TIR1 is expressed in sheath cells, but not in the head neurons, intestine, hypodermis or germ line. They also showed images to suggest that lim-7p::TIR1 does not deplete DAF-2::AID::mNG in the head neurons, germ line and tail neurons.

While we cannot guarantee that the lim-7p::TIR1 line is only expressed in the somatic sheath cells, it is not obviously expressed in other tissues. In our revised manuscript, we have also quantified DAF-2::AID::mNG FI in the germ line in this background and can confirm that DAF-2 is not depleted. We did the same for the ges-1p::TIR1 strain (intestine depletion) for both DAF-2::AID::mNG and DAF-16::GFP::AID and see no germline depletion.

In quantifying DAF-2::AID::mNG germline FI for these experiments, we also repeated the measurements for sun-1p::TIR1 and eft-3p::TIR1 and included the original DAF-2::AID::mNG strain, with no TIR1 driver, as a control. Based on this new data, we can now say that in eft-3p::TIR1 DAF-2::AID::mNG animals, germline DAF-2 is not depleted, which strengthens our conclusion that somatic DAF-2 determines the duration of mitosis in GSPCs. We have included this new data in S4B-C Fig.

2. The authors state in the Methods section that for live-cell imaging, larvae were raised on standard NGM plates seeded with E. coli strain HT115, which produces more uniform developmental timing and a more consistently high number of GSPC divisions. However, the Han lab (Chi et al., Genes and Development 2016) has shown that the commonly used HT115 E. coli strain carries a mutation in cytR and that this mutation makes the strain rich in nucleosides U/T. We do not know if this strain affects all mutants similarly. It strongly suppressed the phenotype of a double mutant of cdd-1 and cdd-2, two cytidine deaminases (Chi et al., Genes and Development 2016). It is possible that specific mutants are rescued by the feeding of this strain rather than others, or their phenotype enhanced. This could certainly explain the conflicting lack of phenotype of rsks-1 mutants also observed in this report. Thus, for live-cell imaging, the authors should still show the results of the analysis with OP50 E. coli.

Response: We agree with the reviewer that gene-diet interactions could have explained the lack of a phenotype that we saw for *rsks-1(ok1255)* and had mentioned this possibility in describing the result in our original manuscript. To address the reviewer's comment, we have repeated this experiment on OP50 and see that GSPC mitosis is not delayed and the PZ seems

normal in terms of number of nuclei and mitotic index. Based on this, we do not think that our use of HT115 as a food source is the reason for the discrepancy between our results and those of Korta et al. (2012) (ref 21). However, in their paper, they outcrossed the *ok1255* allele 8 times into N2, whereas we took the original strain (RB1206) and crossed it into our marker strain (ARG50), without additional backcrosses. It is possible that this difference in genetic background is what underlies the difference in our results and this is what we propose in our revised manuscript. Since the main focus of this manuscript is *daf-2*, we have not pursued this further.

We also tested whether the *daf-2* phenotype was influenced by HT115, by repeating our living cell-imaging on *daf-2(e1370)* animals raised on OP50 and saw a very similar result to what we found with HT115 as a food source. It is not feasible for us to redo our candidate screen on OP50, but since changing to OP50 did not affect the phenotype of *rsks-1(ok1255)* or *daf-2(e1370)*, and since, except for *rsks-1(ok1255)*, the alleles that we tested showed the expected phenotypes in terms of number of PZ nuclei and mitotic index on HT115, we do not think that our choice of HT115 as a food source compromises our results.

Data for *rsks-1(ok1255)* and *daf-2(e1370)* raised on OP50 are shown below.

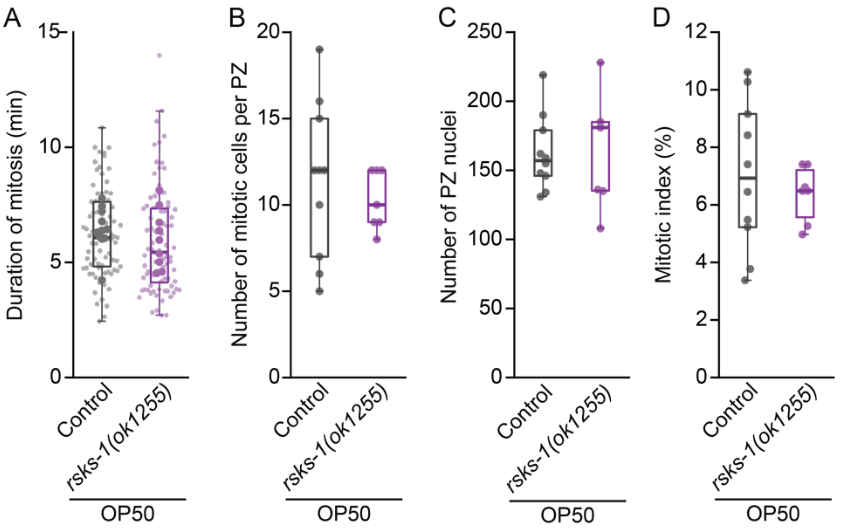

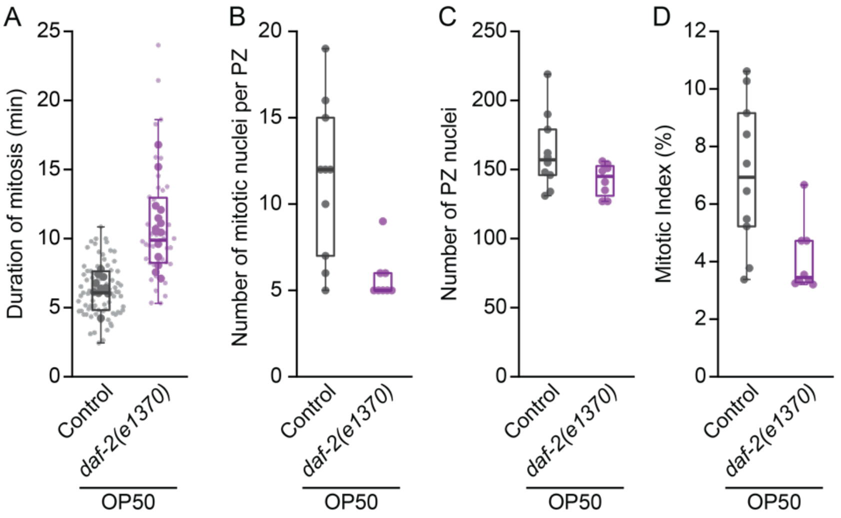

3. A major finding of the report is that DAF-2 and DAF-16 are required in somatic tissues, such as intestine and sheath cells for the timing of the germline mitotic divisions. However, Michaelson et al. had provided rescue experiments and mosaic analysis to show that DAF-16 was required in the germline. Their conclusion was that daf-2, daf-16, daf-18, and hcf-1 were required in the germline primarily, "with a possible additional minor contribution from the soma." In addition, Michaelson et al reported that the ablation of the SS cells, the precursor cells to the sheath and spermatheca, in daf-16(+) and daf-16(-), by RNAi, made no difference, suggesting that DAF-16 in the sheath cells was not a source that promoted germline proliferation. An interesting finding from this report by Cheng et al is that nuclear enrichment of DAF-16/FOXO in the germ line did not appear to affect mitotic timing. These results from Cheng et al. are provocative, however at the very least, they need to expand their discussion of the conflicting results and provide some possible explanations.

Response: We agree with the reviewer that our results were potentially unexpected based on Michaelson et al. (2010), which concluded that larval GSPC cell cycle timing is determined by

germline-autonomous insulin signaling. However, a key difference between our experiments and those of Michaelson et al. is that we are looking at delays in mitosis, while they were looking at phenotypes related to cell cycle progression (number of PZ nuclei and mitotic index (or brood size for their mosaic analysis)), which they attribute to a longer duration of G2. We only assayed these phenotypes for mutant strains (e.g. *daf-2(e1370)* or *daf-16(mu86); daf-2(e1370)*) and there our results align well with Michaelson et al. – in *daf-2* mutants there are fewer PZ cells and a lower mitotic index and these phenotypes require *daf-16* and *daf-18*.

In response to comments from Reviewers 1 and 2 (see above), we have revised our manuscript to clarify the distinction between mitotic delays and cell cycle progression/proliferation in GSPCs, which we hope also makes the relationship between our study and that of Michaelson et al. clearer. We believe our data show convincingly that the duration of GSPC mitosis is determined by DAF-2-to-DAF-16 insulin signaling outside of the germ line, with the intestine being a major site of pathway activity. Michaelson et al. propose that germline autonomous insulin signaling regulates GSPC cell cycle progression during larval development.

The only data we presented in our original manuscript that directly conflicts with that of Michaelson et al., was mentioned in the Discussion, where we raised the question as to whether different anatomical sites of insulin signaling activity affect GSPC cell cycle versus mitotic timing. We mentioned that in our DAF-2 AID experiments, while we did not directly measure the number of PZ nuclei or the mitotic index (the necessary markers are not present in these strains), we did measure the number of mitotic cells per distal gonad arm. *daf-2(e1370)* animals have fewer mitotic cells per distal gonad arm. Depleting DAF-2 with the eft-3p::TIR1 (soma, see response to point 1) or ges-1p::TIR1 (intestine) also leads to fewer mitotic cells per distal gonad arm. Depleting DAF-2 with other drivers, including sun-1p::TIR1 (germline) and lim-7p::TIR1 (somatic sheath) does not.

These results suggests that DAF-2 may not be required in the germ line for larval GSPC proliferation. Alternatively, low levels of germline DAF-2 may be sufficient to sustain normal proliferation. However, the fact that we see fewer cells dividing when DAF-2 is depleted in the soma or in the intestine, suggests somatic tissues may play a larger role than previously appreciated. Tools to properly assess whether genes were required germline autonomously were not available when Michaelson et al. was published. Instead, the authors used RNAi experiments in the *rrf-1* background where RNAi is intact in the germline, but somatic RNAi is less efficient, to assess whether *daf-2* was required in the germline. However, one of the somatic tissues in which RNAi is still effective in *rrf-1* mutants is the intestine (Kumsta and Hansen, 2012, https://doi.org/10.1371/journal.pone.0035428).

We cannot directly address the rescue and mosaic experiments described in Michaelson et al., which they use as evidence that *daf-16* also acts in the germline to regulate GSPC proliferation, as none of our experiments tested this. Our data suggest that *daf-16*, like *daf-2*, acts in the intestine to affect the duration of GSPC mitosis. We performed these experiments using *daf-2(RNAi)* which delays GSPC mitosis, but does not lead to a strong phenotype in terms of GSPC

proliferation. As a result, our data does not allow us to determine whether depletion of DAF-16 in the intestine rescues GSPC proliferation as well as mitotic duration.

We think the model of Michaelson et al. may need to be revisited with new tools. However, we feel that it is outside of the scope of our paper to address this further. We have revised the relevant section of our Discussion to clarify the distinction between our work at that of Michaelson et al. and to further discuss the alternative hypothesis that the regulation of mitosis and the regulation of cell cycle progression/proliferation are separable phenotypes which may rely on different sites of insulin pathway activity. We have also removed the data mentioned above where we counted the number of mitotic cells per gonad arm when DAF-2 was depleted using different TIR1 drivers as in revising our manuscript to focus it on the relationship between insulin signaling and mitosis it was no longer relevant.

4. The authors describe that strains carrying TIR1 expressed from different promoters and the DAF-2::AID::mNG entered dauer when raised from L1 on 1mM K-NAA plates, but that animals raised in control (no K-NAA) showed a "weak daf-2 phenotype." Have the authors tested other existing DAF-2::AID strains? There are at least two other strains available in the literature [Venz et al., Elife 2022; Roy et al., Aging Cell 2022]. A different AID-degron tagged strain would perhaps allow for the analysis of DAF-2.

Response: We thank the reviewer for the suggestion. We have observed that some TIR1 drivers (particularly eft-3p) are leaky and induce some depletion of AID-tagged proteins even in the absence of auxin. This is a known limitation of the system (e.g. Negishi et al. 2022, ref 107, Schiksnis et al. 2020, ref 108) and unlikely to be solved by using a different AID-tagged version of DAF-2, unless engineered using the strategy in Negishi et al. The AID-tagged versions of DAF-2 in Venz et al. and Roy et al. still use the original AID tag and we have no reason to think they would behave differently than the AID-tagged DAF-2 from Zhang et al. (2022) (ref 61) that we used here. These strains are also less readily available (they are not at the CGC) and to redo our experiments with them would require considerable strain construction.

We also do not think that the challenges of working with DAF-2::AID limited our experiments. For the eft-3p, we took advantage of the 'leakiness' to assess GSPC mitosis in animals were DAF-2 was partially depleted in the soma. Since germline DAF-2 expression is not reduced in these worms (see new S4B-C Fig.), this allowed us to test whether DAF-2 was required in the soma. For the experiments with ges-1p, myo-3p and dpy-7p, we transferred larvae to auxin plates after the dauer commitment stage, and this allowed us to assess mitosis in late L4 larvae, as in other experiments.

5. In the methods, the authors describe that synchronized animals were obtained by hypochlorite treatment, however this may introduce added stress. Are the authors sure that hypochlorite treatment has no effect on the process they study? A better way to synchronize animals is to gather L1 as they hatch off, see McCarter et al., 1997.

Response: We agree with the reviewer and have moved away from hypochlorite treatment for this reason. Hypochlorite treatment was only used for L1 synchronization for some of the imaging performed as part of our candidate screen (Fig 1) and for the *(daf-2)RNAi* experiments in S3B and S4D. Most data was collected from larvae synchronized by a short egg collection (3-5 adults were allowed to lay eggs for 2-3 hours), which is similar to the approach in McCarter et al. except that we let all embryos hatch and select late L4s by vulval morphology and size ~3 days later. We have made this clearer in our revised Methods section.

In response to the reviewer's comment, we checked whether the duration of mitosis was different across control worms (strain ARG50) synchronized by the different methods. This data is shown below. "Direct transfer" refers to L1s picked from recently starved NGM/OP50 plates. The fourth column is the control data from our screen which was a combination of egg collection and hypochlorite treatment. None of the groups are statistically different from one another which suggests the duration of GSPC mitosis, at least in a control strain, is not sensitive to the method of synchronization.

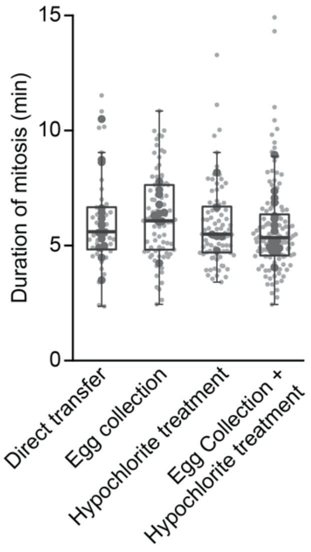

Minor points:

On page 8, line 147, the authors should define NEBD. They do define it in the figure legends, but this is the first time NEBD is used in the body of the results section.

Fixed. Thank you.
